# A Combination of Radiosurgery and Soluble Tissue Factor Enhances Vascular Targeting for Experimental Glioblastoma

**DOI:** 10.1155/2013/390714

**Published:** 2013-11-06

**Authors:** Jian Tu, Zhiqiang Hu, Zhongbin Chen

**Affiliations:** ^1^Australian School of Advanced Medicine, Macquarie University, 2 Technology Place, North Ryde, Sydney, NSW 2109, Australia; ^2^Department of Neurosurgery, The 9th Medical Clinical College of Beijing University, Beijing 100850, China; ^3^Department of Electromagnetic and Laser Biology, Beijing Institute of Radiation Medicine, Beijing 100038, China

## Abstract

Radiosurgery for glioblastoma is limited to the development of resistance, allowing tumor cells to survive and initiate tumor recurrence. Based on our previous work that coadministration of tissue factor and lipopolysaccharide following radiosurgery selectively induced thrombosis in cerebral arteriovenous malformations, achieving thrombosis of 69% of the capillaries and 39% of medium sized vessels, we hypothesized that a rapid and selective shutdown of the capillaries in glioblastoma vasculature would decrease the delivery of oxygen and nutrients, reducing tumor growth, preventing intracranial hypertension, and improving life expectancy. Glioblastoma was formed by implantation of GL261 cells into C57Bl/6 mouse brain. Mice were intravenously injected tissue factor, lipopolysaccharide, a combination of both, or placebo 24 hours after radiosurgery. Control mice received both agents after sham irradiation. Coadministration of tissue factor and lipopolysaccharide led to the formation of thrombi in up to 87 ± 8% of the capillaries and 46 ± 4% of medium sized vessels within glioblastoma. The survival rate of mice in this group was 80% versus no survivor in placebo controls 30 days after irradiation. Animal body weight increased with time in this group (*r* = 0.88, *P* = 0.0001). Thus, radiosurgery enhanced treatment with tissue factor, and lipopolysaccharide selectively induces thrombosis in glioblastoma vasculature, improving life expectancy.

## 1. Introduction

Glioblastoma (GBM) is highly fatal with a median survival time of 7 months [1]. Its rapid growth leads to increased intracranial pressure, which eventually results in death. In terms of years of life lost, the population burden from GBM is the highest of all the malignant cancers [2]. Current treatment for this disease consists of open craniotomy with surgical resection, followed by concurrent or sequential chemoradiotherapy and radiosurgery [3]. Complete surgical excision of GBM is impossible as individual tumor cells can deeply infiltrate adjacent normal brain tissue. GBMs are especially resistant to radiosurgery and chemotherapy and tend to recur after treatment. Recurrent GBM proliferates rapidly due to the loss of multiple cell-cycle inhibitors and increases signaling from multiple growth factor receptors that act through downstream effectors to exert positive effectors on the regulation of the cell cycle [4]. 

Although considerable effort has been invested in the discovery of different approaches that target various aspects of GBM genesis cascade and several agents are in various stages of development or have advanced to clinical trials, it is becoming increasingly apparent that GBM vasculature is an attractive target for therapy because the provision of oxygen and nutrients by a single vessel supports the survival of many tumor cells, as well as provides a main route for metastatic spread [5]. Therapeutic vascular targeting has so far concentrated on either antiangiogenic approaches, which aim to prevent the neovascularisation processes in tumors, or antivascular approaches that aim to cause the selective shutdown of the established tumor vasculature leading to tumor cell death. Selective induction of intravascular thrombosis in the tumor vasculature but not in normal tissue relies on the ability to exploit molecular differences in the luminal surface of endothelial cells lining tumor vessels versus normal vascular endothelial cells.

Compared with the vasculature in normal brain tissue, the GBM vasculature is strikingly chaotic, featuring complex branching patterns and lack of hierarchy [6, 7]. Indeed, it is often difficult to distinguish arterioles and venules, and the occurrence of vascular shunts is common in GBM vasculature [6]. Vessel diameters are irregular, and lengths between branching points are often very long. The result is a high geometrical resistance to blood flow, such that a small decrease in perfusion pressure, which has little effect in normal tissues, can be catastrophic in GBM [8]. Vessel walls in GBM are immature, often with a discontinuous endothelial cell lining, and have poor connections between pericytes and endothelial cells and an irregular, structurally abnormal basement membrane [9]. Endothelial cells in GBM vessels are often irregularly shaped, forming an uneven luminal layer, with loose interconnections and focal intercellular openings [10]. Furthermore, endothelial cells in a normal cerebral vascular system show an absence of Weibel Palade bodies [11]; however, in GBM vessel walls, Weibel Palade bodies can be identified in endothelial cells. Most importantly, the prothrombotic lipid phosphatidylserine (PS) is exposed on the vascular endothelium of all solid tumors, including GBM, but not on endothelium in normal tissues [12–17]. All of these features are also seen in cerebral arteriovenous malformations [11, 18–24], and may represent the key to translating our effective radiosurgery enhanced vascular targeting for cerebral arteriovenous malformations into GBM therapy.

We have previously undertaken studies to develop a radiosurgery enhanced vascular targeting for cerebral arteriovenous malformations, which achieved fast, selective, and sustained intravascular thrombosis of 69% of the capillaries and 39% of medium size vessels of arteriovenous malformations [18]. A rapid and selective shutdown of the capillaries and medium sized vessels in GBM vasculature would significantly decrease the delivery of oxygen and nutrients, thereby reducing cell division and GBM expansion, leading to a decline in intracranial hypertension, and improving life expectancy of patients. 

The objectives of this study were to examine whether a combination of radiosurgery and soluble tissue factor could selectively occlude the capillaries that supply oxygen and nutrients to a mouse model of glioblastoma. This is because of our previous work on cerebral arteriovenous malformations, where a similar approach has achieved rapid and selective thrombosis of 69% of the capillaries and 39% of medium sized vessels. We hypothesize that selective thrombosis of GBM capillaries can be achieved with a single low dose of highly focused radiation precisely delivered to the tumor that induces apoptosis and externalization of phosphatidylserine from the inner leaflet of endothelial cell membrane lipid bilayer, followed by an intravenous injection of soluble tissue factor as the primary initiator of the extrinsic coagulation cascade to bind the exposed phosphatidylserine, causing selective thrombosis of GBM vasculature, reducing blood supply to the tumor, leading to GBM cell death, reducing tumor size and intracranial pressure, and improving life expectancy of GBM animals. 

## 2. Materials and Methods

### 2.1. Chemicals, Reagents, and Cell Culture

All chemicals and purified lipopolysaccharide (LPS) from *Escherichia coli *serotype055:B5 were purchased from Sigma-Aldrich (St. Louis, MO, USA) unless otherwise specified. The 3G4 anti-phosphatidylserine antibody was provided by Prof. Philip Thorpe (University of Texas Southwestern Medical Center, Dallas, TX, USA). Recombinant soluble tissue factor (a kind gift of Diagnostic Stago, Asnières sur Seine, France) was produced by the expression of the extracellular domain of human tissue factor in *Escherichia coli *that was then refolded into soluble form. Anti-mouse von Willebrand factor and caspase-3 antibodies were purchased from Abcam (Cambridge, UK). Alexa Fluor 488 or Alexa Fluor 594 anti-rabbit secondary antibodies were purchased from Molecular Probes (Eugene, OR, USA).

Dulbecco's modified Eagle's medium (DMEM), RPMI1640 medium, fetal calf serum (FCS), phosphate buffered saline (PBS), L-glutamine, and trypsin were purchased from Invitrogen (Carlsbad, CA, USA). The MTT assay kit was purchased from Roche Applied Science (Indianapolis, IN). Immortalized mouse brain endothelial cells (bEnd.3) were purchased from the American Type Culture Collection (ATCC, Manassas, VA, USA), and maintained in high glucose DMEM with 10% FCS as previously reported [25].

### 2.2. Cell Viability Assay Following Irradiation

 To evaluate the effect of radiation on cell viability and select irradiation dose,  bEnd.3 cells were examined by MTT assay following irradiation as previously reported [25, 26]. Briefly, bEnd.3 cells were seeded in 96-well plates coated with Haemaccel (Boehring Pharma, Amsterdam, Netherlands) at a density of 8 × 10^3^ for 24 hours. When cells were 40–60% confluent, the cell medium was replaced by RPMI1640 phenol red free medium supplemented with 10% FCS and 10 *μ*L/mL L-glutamine. The cell cultures were then exposed to radiation delivered by an orthovoltage X-ray generator (Philips RT100, Royal Philips Electronics, Amsterdam, Netherlands) with a dose of 6, 12 or 20 Gray. Sham controls were treated identically but were not exposed to radiation. 

The 3-(4,5-dimethylthiazol-2-yl)-2,5-diphenyltetrazolium bromide (MTT) was used to determine an index of cellular viability and mitochondrial metabolic activity. MTT was prepared at 5 mg/mL in RPMI1640 phenol red free medium. The assay activity was determined at 6, 12, 24, and 72 hours after irradiation and performed in triplicate. At each of these time points, 10 *μ*L of MTT was added to each well and incubated for 4 hours at 37°C. One hundred *μ*L of formanzan lysis was then added to each well and incubated for another 4 hours at 37°C. The absorbance of the solubilized formanzan was measured at 570 nm using a microplate reader (iMark, Bio-Rad, Hercules, CA, USA). The viability of cells in irradiation sham controls was considered as 100%. 

### 2.3. GBM Mouse Model

The GL261 mouse glioma cell line of C57Bl/6 origin was used as seeding cells for GBM that has growth characteristics similar to human GBM [27]. The GL261 glioma cells were maintained in RPMI 1640 medium supplemented with 10% FBS in a humidified incubator gassed with 5% CO_2_ at 37°C until reaching 70% confluence. Animal experimentation was approved and performed in accordance with the guidelines of the corresponding institutional Experimental Animal Care and Ethics Committee and the Code of Practice for the Care and Use of Animals for Scientific Purposes [28]. Eighty-two male C57Bl/6 mice (7-8 weeks old, 20 ± 1 g) were housed in a parasite-free environment. Bedding was changed twice a week, and food and water were provided ad libitum. Animals were allowed to acclimatize to new surroundings before the experiment began. According to our experimental design, the minimal number of animals required was used. Mice were randomly divided into 5 groups.

The mouse was placed in a Perspex induction chamber. Anaesthesia was induced with a mixture of isoflurane (4%) and oxygen (2 L/min). The mouse was then transferred to the operating rig, upon which general anaesthesia was maintained with the mouse self-ventilating isoflurane via a nose cone. The depth of anaesthesia was assessed using the respiratory rate and by checking the hind limb withdrawal to pain with the use of a blunt forcep. No procedure was commenced until there was a consistent absence of response to pain. Observations of respiratory rate, rectal core temperature, and pain response were recorded every 5 minutes for the first 20 minutes and fifteen minutely thereafter. For the duration of the procedure, there was continuous monitoring of rectal core temperature and of oxygen saturation via transcutaneous pulse oximetry on a hind limb. A heating blanket was used for the duration of the procedure.

The GL261 glioma cells were implanted into the right striatum of the mouse. The mouse was placed in the Kopf rodent stereotactic apparatus (Tujunga, CA, USA) under general anaesthesia. The operation site was shaved and prepared with povidone-iodine. The procedure was performed in a sterile field using aseptic technique. A burr hole (0.18 mm in diameter) was placed at the stereotaxic coordinates: AP = 0.5 mm, ML = 1.8 mm (from bregma). A Hamilton syringe was advanced to a depth of 2.35 mm from the cortical surface, and 4 *μ*L of medium containing 2 × 10^4^ GL261 glioma cells were injected. Subsequently, the bony calvarium was sealed using a droplet of bone wax to prevent reflux, and the skin was sutured. After the procedure, observations were carried out daily. Observations included weight, assessment of motor function, behaviour, and wound health. Ten days after implantation when mouse started to exhibit visible tumor (2.8 ± 0.7 mm^3^), the mouse received radiosurgery enhanced vascular targeting treatment.

### 2.4. Radiosurgery and Vascular Targeting

Anaesthesia was initially induced with a mixture of isoflurane (4%) and oxygen (2 L/min). Ketamine (40 *μ*g/g) and midazolam (1.5 *μ*g/g) were given intramuscularly 20 minutes prior to radiosurgery. Once the righting response was lost, the mouse was given an additional dose of ketamine (20 *μ*g/g) and midazolam (0.75 *μ*g/g) to ensure an adequate duration of anaesthesia. Whilst sedated, animals freely breathed oxygen (2 L/min) via a nose cone. The depth of anaesthesia was assessed using the respiratory rate and by checking the hind limb withdrawal to pain. Routine observations were made of respiratory and heart rates. 

Radiosurgery was delivered using a modification of previously reported protocol [18]. Once sedated, the mouse was placed on the stage attached to the head ring of the X-Knife linear accelerator (Radionics, Burlington, MA, USA). Correct positioning of the mouse relative to the planned treatment location was confirmed by ensuring that the site of implantation of GL261 glioma cells was placed at the intersection of the three targeting laser beams that designate the centre of the radiation delivery arcs. The same treatment plan was applied for all mice, giving a maximal dose of 6 Gray to the GBM in the subcranial region. The sham radiation controls were treated identically but without irradiation.

Twenty-four hours following radiosurgery, mice received intravenous administration of either sterile phosphate buffered saline (PBS) or lipopolysaccharide (LPS) and soluble tissue factor (TF); or they received LPS and TF 24 hours following sham radiosurgery. The LPS was given as a dose of 0.1 *μ*g/g body weight. The dose of TF was 0.4 *μ*g/g body weight. The LPS and TF were suspended in 1 mL 0.9% sterile PBS. 

### 2.5. Histology and Immunohistochemistry

Immediately following perfusion, brain, heart, lungs, and liver were harvested, embedded in paraffin for histology and in tissue freezing medium (ProSciTech, QLD, Australia) with liquid nitrogen for immunohistochemistry. Hematoxylin and eosin (H&E) or Martius Scarlet blue staining was performed as previously described [11, 18, 24]. Briefly, sections were deparaffinized with xylene, rehydrated in alcohol, and then placed in hematoxylin for 5 minutes. Slides were differentiated in 2% hydrochloric acid alcohol, bathed in lithium carbonate for 15 seconds to perform bluing, and counterstained with eosin for 2 minutes. Slides were dehydrated with ethanol and washed in xylene before being coverslipped and examined using a microscope (Axioplan 2, Carl Zeiss, Germany), and imaging data were acquired by a digital camera (AxioCam MRm, Carl Zeiss, Germany) using Axiovision software (Carl Zeiss, Germany). 

Sections were stained immunohistochemically as previously described [18, 20–23]. Briefly, sections were washed in PBS at 37°C to remove tissue freezing medium. Nonspecific binding was blocked by 10% horse serum in PBS. Primary antibodies were applied and incubated at 4°C overnight. Sections were washed in PBS, incubated with secondary antibodies for 2 hours in dark, coverslipped, and examined using a confocal microscope (Leica SP5, Germany), and imaging data analyzed using Leica LAS AF software. Fluorescence intensity units (FIU) were corrected using primary antibody controls. The FIU has been quantified as mean gray value. 

### 2.6. Data Analysis

 Data are expressed as means ± SE (number of mice). Statistical difference between groups was determined using the unpaired two-tailed *t*-test. When there were more than two groups, differences were analyzed using analysis of variance if the variances were equal and the Mann-Whitney nonparametric test if variances were unequal [29]. Linear regressions were calculated using a statistical computer package, Number Cruncher Statistical Systems [29]. A value of *P* < 0.05 was considered statistically significant.

## 3. Results

### 3.1. Cell Viability

**Figure 1:**
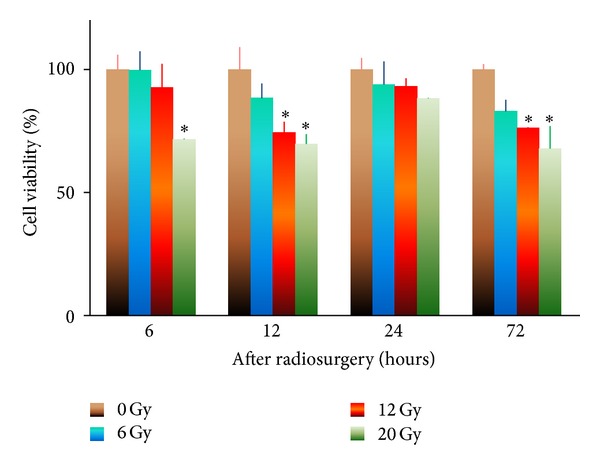
Effect of radiosurgery on cell viability. Mitochondrial metabolic activity of bEnd.3 cells was determined by MTT assay as an indicator of cell viability following radiosurgery at 0, 6, 12, and 20 Gray. *P < 0.05 paired comparison at the same time point.

 Radiation effect on bEnd.3 cell viability was assessed as shown in Figure 1. The cell viability of sham irradiation controls was considered as 100%. At 6 hours after irradiation, a range of radiation dosages from 0 to 20 Gray had no significant effect on bEnd.3 cell viability (*r* = −0.9213,  *P* = 0.0787). At 12, 24, and 72 hours after irradiation, there was a negative correlation between radiation dose and bEnd.3 cell viability (*r* = −0.9675,  *P* < 0.04; *r* = −0.9623,  *P* < 0.04; *r* = −0.9649,  *P* < 0.04, resp.), suggesting that the cell viability declined with increasing radiation dosage over a period of 12–72 hours after irradiation. Six Gray irradiation did not induce any significant decline in bEnd.3 cell viability. Twelve Gray radiation resulted in deduction of cell viability 12 and 72 hours following irradiation. Twenty Gray radiation reduced cell viability significantly. Therefore, 6 Gray was selected as radiation dose for further study.

### 3.2. Apoptosis

**Figure 2:**
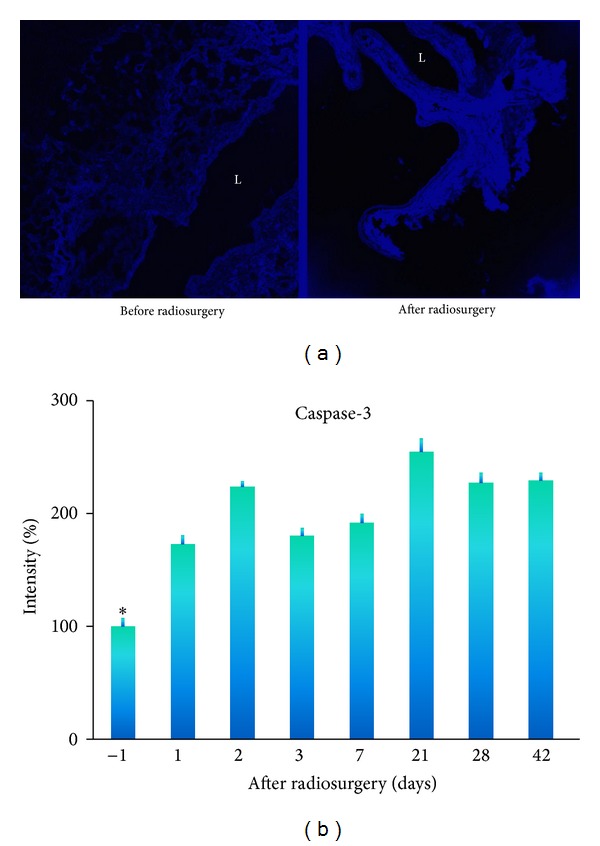
Apoptosis following radiosurgery. Caspase-3 was examined immunohistochemically before and after radiosurgery at 6 Gray. The intensity of immunofluorescence of vessels was quantified using a confocal microscope. L, lumen. Data expressed as means ± SE of 4 mice at each time point. *P < 0.05 paired comparison between before (−1 day) and after radiosurgery at different time points.

Caspase-3 is a key enzyme in apoptotic signaling pathway and is selected as a marker for apoptosis. The levels of caspase-3 expression in GBM capillaries over a period of 42 days after radiosurgery at 6 Gray were shown in Figure 2. There was a significant upregulation of caspase-3 expression in GBM capillaries after irradiation. The levels of caspase-3 overexpression were 72% at 1 day after irradiation and peaked at 154% at 21 days after radiation. 

### 3.3. Externalization of Phosphatidylserine

**Figure 3:**
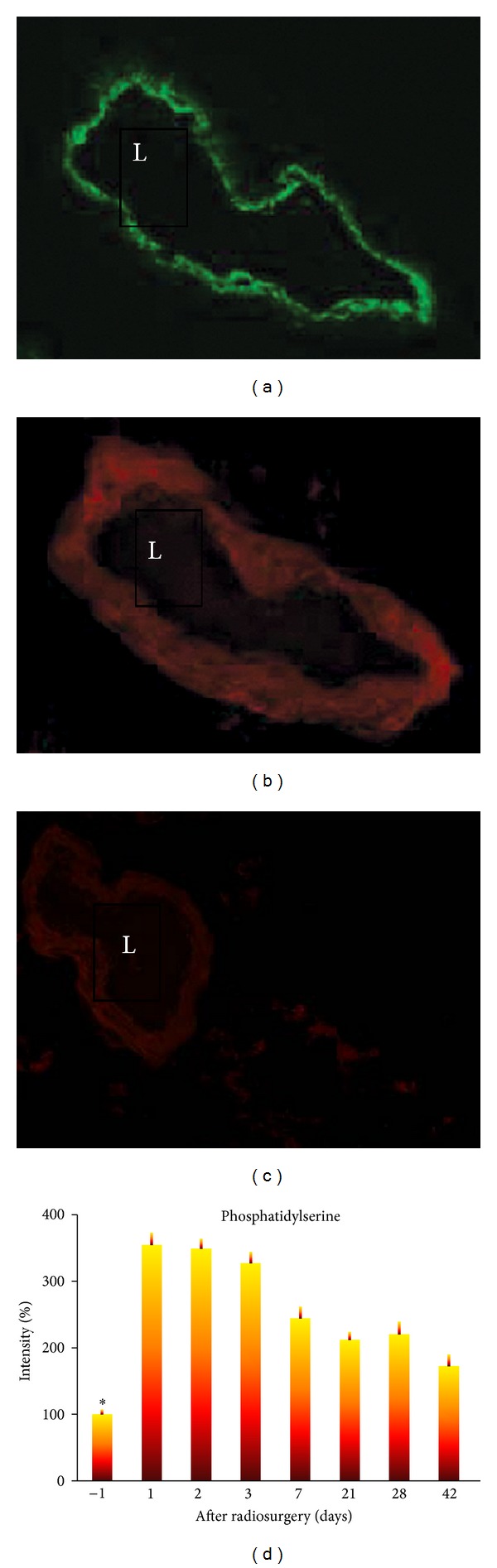
Radiosurgery induced externalization of phosphatidylserine from the surface of endothelial cells of GBM capillaries. (a) Positive immunofluorescent staining of specific endothelial cell marker, von Willebrand factor, on the surface of endothelial cells of GBM capillaries in mice that received radiosurgery. (b) Positive immunofluorescent staining of phosphatidylserine on the surface of endothelial cells of GBM capillaries in mice that received radiosurgery. (c) Weak immunofluorescent staining of phosphatidylserine on the surface of endothelial cells of GBM capillaries in mice that received sham radiosurgery. (d) Time course of immunofluorescent intensity of phosphatidylserine on the surface of endothelial cells of GBM capillaries in mice that received sham radiosurgery (−1 day) or radiosurgery over a period of 42 days. There is a negative correlation between time and the immunofluorescent intensity of phosphatidylserine on the surface of endothelial cells of GBM capillaries in mice that received radiosurgery over a period of 42 days (*r* = −0.9,  *P* < 0.006). *P < 0.001 paired comparison between before (−1 day) and after radiosurgery at different time points. L, lumen.

 The exposure of phosphatidylserine (PS) on the outer cell membrane is essential to regulate the activity of tissue factor. Radiosurgery induced apoptosis in vascular endothelial cells, resulting in externalization of phosphatidylserine on the cell surface of the lumen of GBM capillaries in mice that received radiosurgery as shown in Figure 3. The immunofluorescent intensity of phosphatidylserine increased by 2.5-fold on the surface of endothelial cells of GBM capillaries 24 hours following radiosurgery compared with that in sham irradiation controls (*P* < 0.001). These externalised phosphatidylserine molecules offer themselves as binding targets of soluble tissue factor in blood circulation. 

### 3.4. Thrombosis

**Figure 4:**
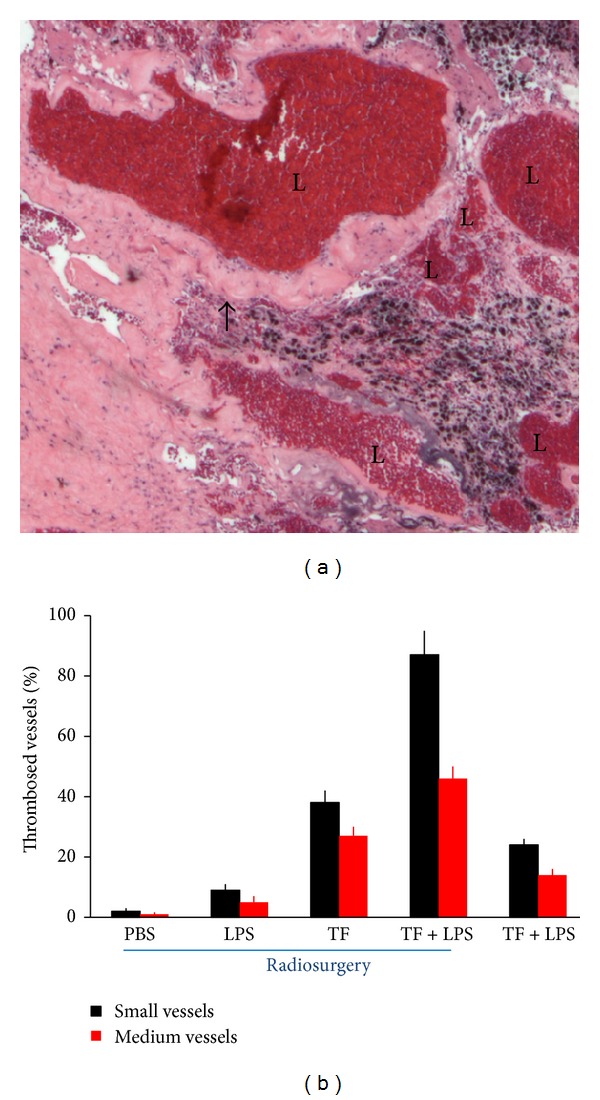
A combination of radiosurgery and soluble tissue factor induces thrombosis. (a) GBM capillaries completely occluded by thrombi. There is loss of cellularity and hyaline transformation of the wall matrix (*arrowhead*) (H&E staining). L, obliterated lumen. (b) Thrombosis rates in small (<50 *μ*m in diameter) and medium (50–200 *μ*m) vessels 17 days after treatment. PBS, phosphate buffered saline; LPS, lipopolysaccharide; TF, soluble tissue factor; TF + LPS, coadministration of soluble tissue factor and lipopolysaccharide. Data were obtained from vessels stained using Martius Scarlet blue and expressed as means ± SE of 10 mice per group. *P* < 0.05 paired comparison in small vessels or medium vessels between treatment groups.

Circulated soluble tissue factor binds to externalised phosphatidylserine on the cell surface of the lumen of GBM vessels, resulting in selective thrombosis of GBM medium and small vessels as shown in Figure 4. There was significant higher thrombotic rate in small vessels (<50 *μ*m in diameter) than medium vessels (50–200 *μ*m in diameter) of GBM (*P* < 0.05) in all treatment groups. In sham irradiation controls, a combination of soluble tissue factor and lipopolysaccharide induced 24 ± 2% thrombosis in small vessels of GBM and 14 ± 2% thrombosis in medium vessels of GBM. There was 41.6 ± 2% higher thrombosis in small vessels than medium vessels of GBM (*P* < 0.01). Following radiosurgery, a combination of soluble tissue factor and lipopolysaccharide resulted in 87 ± 8% thrombosis in small vessels of GBM and 46 ± 4% thrombosis in medium vessels of GBM. There was 47.1 ± 6% higher thrombosis in small vessels than medium vessels of GBM (*P* < 0.01). Soluble tissue factor induced 38 ± 4% thrombosis in small vessels of GBM and 27 ± 3% thrombosis in medium vessels of GBM. There was 28.9 ± 4% higher thrombosis in small vessels than medium vessels of GBM (*P* < 0.05). Lipopolysaccharide produced 9 ± 2% thrombosis in small vessels of GBM and 5 ± 2% thrombosis in medium vessels of GBM. There was 44.4 ± 2% higher thrombosis in small vessels than medium vessels of GBM (*P* < 0.05). Phosphate buffered saline generated 2 ± 1% thrombosis in small vessels of GBM and 1 ± 1% thrombosis in medium vessels of GBM. There was 50.0 ± 1% higher thrombosis in small vessels than medium vessels of GBM (*P* < 0.01). This baseline level of radiosurgery induced site-specific thrombosis is most likely associated with a normal level of thrombogen, including tissue factor that exists in blood circulation. Logistic regression analysis of the experimental data from all treatment groups demonstrated significant associations between development of thrombi and treatment with radiation, soluble tissue factor, or phosphatidylserine (*P* < 0.005). No intratumoral bleeding was observed in any treated groups. No mice were found to have thrombi in the vasculature of normal brain, lung, heart, or liver nor was there evidence of infarction in these organs.

### 3.5. *In Vivo* Study

**Figure 5:**
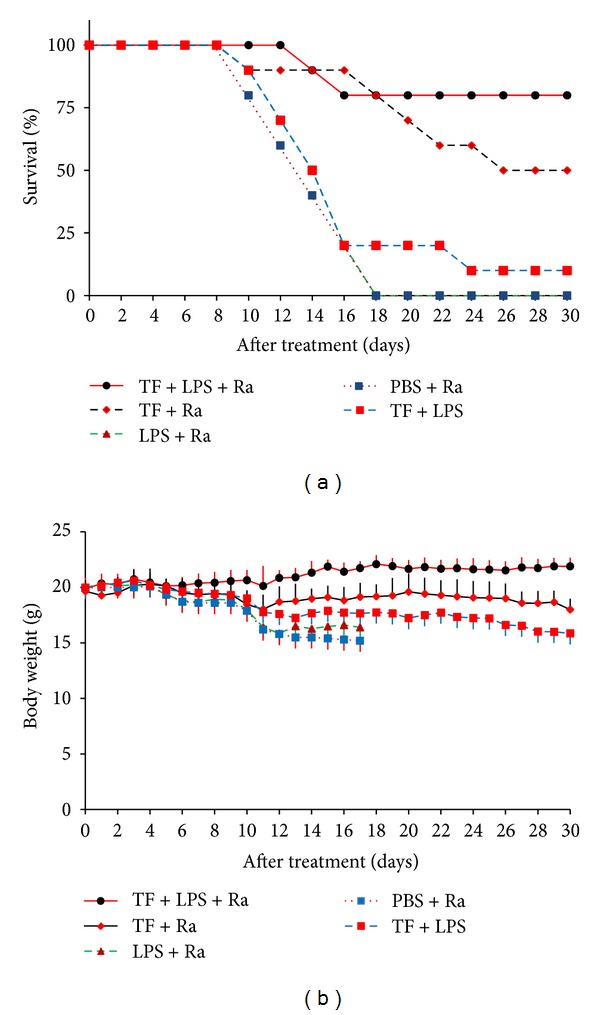
Survival rate and body weight of GBM mice. Forty GBM mice were randomly divided into 5 groups and received coadministration of soluble tissue factor and lipopolysaccharide (TF + PLS) or a combination of radiosurgery and soluble tissue factor plus lipopolysaccharide (TF + PLS + Ra), soluble tissue factor (TF + Ra) lipopolysaccharide (PLS + Ra), and saline (PBS + Ra), respectively. Animals were i.v. injected with different agents 24 hours following a 6 Gray radiosurgery. (a) TF + PLS + Ra treatment delivered the highest survival rate of GBM mice. TF + Ra group produced a higher survival rate than TF + LPS group. No animal survived more than 17 days in PBS + Ra and PLS + Ra groups. (b) Body weight of TF + PLS + Ra group was increased with time over a period of 30 days (*r* = 0.8783,  *P* < 0.0001). Animal body weight in TF + Ra, TF + LPS, LPS + Ra, and PBS + Ra was declined with time (*r* = −0.5616,  *P* < 0.0005; *r* = −0.9453,  *P* < 0.001; *r* = −0.9322,  *P* < 0.001; *r* = −0.9578,  *P* < 0.001, resp.).

 The potential effect of radiosurgery enhanced tissue factor vascular targeting for glioblastoma was assessed in GBM mice. The animal survival rate and survival time over a period of 30 days were shown in Figure 5. No mice in the PBS and phosphatidylserine groups survived beyond 17 days after irradiation. The final survival rates of GBM mice that received a combination of radiosurgery and a low dose of soluble tissue factor and phosphatidylserine, a combination of radiosurgery and soluble tissue factor, and coadministration of soluble tissue factor and phosphatidylserine on day 30 were 80%, 50% and 10%, respectively. Compared with the PBS and phosphatidylserine groups, both animal survival time and survival rate significantly improved in mice that received a combination of radiosurgery and a low dose of soluble tissue factor and phosphatidylserine (*P* < 0.001), a combination of radiosurgery and soluble tissue factor (*P* < 0.001), and coadministration of soluble tissue factor and phosphatidylserine (*P* < 0.05). Compared with the group that received coadministration of soluble tissue factor and phosphatidylserine but without radiosurgery, the animal survival rates were significantly higher in mice that received a combination of radiosurgery and a low dose of soluble tissue factor and phosphatidylserine or a combination of radiosurgery and soluble tissue factor (*P* < 0.001). Compared with the group that received a combination of radiosurgery and soluble tissue factor but without phosphatidylserine, the animal survival rate was significantly higher in mice that received a combination of radiosurgery and a low dose of soluble tissue factor and phosphatidylserine (*P* < 0.01).

The body weight of irradiated GBM mice that received coadministration of soluble tissue factor and phosphatidylserine was found to increase with time over a period of 30 days. We observed a positive correlation between animal body weight and time in this group with the correlation coefficient (*r*) being 0.8783 (*P* < 0.0001). In contrast, animal body weight in all other groups declined with time over a period of 17 or 30 days (*P* < 0.0005). These results demonstrate that a combination of radiosurgery and coadministration of soluble tissue factor and phosphatidylserine offers the potential to improve life expectancy and quality of mice suffering from glioblastoma.

## 4. Discussion

Based on our previous work on brain arteriovenous malformations and the described similarities in vascular structure in gliomas, we hypothesized that vascular targeting of tumor vessels would be achieved with a single dose of radiation precisely delivered to the tumor inducing apoptosis of immature glioma vascular endothelial cells and externalise PS (a co-factor of tissue factor). This would be followed with a single intravenous injection of tissue factor that binds to PS exposed on the outer leaflet of the bilayer, triggering the cascade of events in selective thrombosis of the tumor vasculature. This strategy produces the following two phases of effects. 

### 4.1. The Direct Effects of Radiation

 Although irradiated cerebral endothelial cells maintained reasonable viability (Figure 1), they are rendered incapable of proliferation in a process similar to senescence [30]. These cells remain metabolically active just as we observed in MTT assay (Figure 1) but will be incapable of further division [31]. It has been suggested that irradiation induces potentially lethal double-stranded breakage of DNA, but these breaks may be stabilized by a process known as telomere capture. Telomeres are short, highly repetitive DNA sequences that shield the ends of chromosomes from degradation and end-to-end fusions. After each cell division telomeres become shorter than they were before and upon reaching a critical length, cells enter G_1_ arrest and are unable to undergo further proliferation [30]. If these senescent cells are forced to divide further, their telomeres continue to shorten. When telomere length is less than 1 kb, no further division is possible and a necrotic-like cell death results. Telomere capture involves the transfer of telomeres from normal chromosomes to the broken chromosomes. This capture of the normal telomeres will stabilize the damaged DNA while hastening the induction of senescence [30]. This explains that there was no new blood vessels observed to revascularize the tumor in the group treated with a combination of radiosurgery and soluble tissue factor plus lipopolysaccharide.

When radiation is precisely delivered to the lesion, it causes damage to the vessel walls and increases permeability of the vasculature [24]. Leakage of plasma proteins from the vessel reduces the oncotic pressure differential between the inside and outside blood vessels, disturbing the water balance and potentially causing a transient increase in interstitial fluid pressure, which might be sufficient to reduce vessel diameter [32]. The loss of fluid also leads to an increased hematocrit to increase viscous resistance to blood flow. It is also possible that the longitudinal pressure differential along the vessel decreases, thereby further reducing flow and facilitating thrombosis [32]. Radiation also causes proliferation of smooth muscle cells and enlargement of endothelial cells *in vivo *[24]. This is sufficient to shrink vessel diameter and reduce the size of thrombi required to occlude vessels. 

The vasculopathy induced by radiation is both time and dose dependent, with both longer times after exposure and larger doses having more severe lesions [33]. The individual changes induced by radiation in the vasculature are not unique, but the combination of effects on the vasculature and surrounding stroma is often characteristic. The capillaries are the most sensitive components of the vascular tree to radiation damage [34–37]. There is considerable heterogeneity of capillary response to radiation with respect to the time course and dose required for significant alterations to occur; however, the pattern of changes is similar among different tissues. The available evidence suggests that endothelial damage of capillaries is a very important mechanism of delayed radiation injury [33, 38].

Antivascular therapy has been reported that occlusion of blood vessels supplying tumors in rodents led to tumor regression [39]. The vascular targeting approach affects pre-existing blood vessels with the following advantages.

- In tumors, a single capillary provides blood to hundreds or thousands of tumor cells. If the capillary is occluded at one point, the flow of blood to these cells is ceased [40–42].
- The occlusion of tumor vasculature may only need to be transient, with *in vivo *studies indicating that greater than 99% of tumor cells can be killed during a 2-hour period of ischemia [42].
- The endothelial cells in tumor vasculature are freely accessible to targeting molecules that are delivered intravenously [39, 40, 42–44].
- The endothelial cells within tumor vasculature are normal diploid cells. Unlike tumor cells, these endothelial cells are not expected to acquire genetic mutations that develop radiation resistance [40, 42].
- Unlike antiangiogenesis agents that require continuous administration, vascular targeting agents require only intermittent administration. In preclinical studies, it appears that vascular targeting agents are capable of shutting down tumor vascular trees that supply blood to as much as 95% of a solid tumor, resulting in widespread central necrosis [42, 45, 46].

Therefore, vascular targeting strategy was applied for experimental GBM therapy.

### 4.2. Thrombosis Induced by Administration of Tissue Factor

 The prothrombotic lipid phosphatidylserine (PS, a co-factor of tissue factor) is normally intracellular but becomes exposed on the luminal surface of vascular endothelium in tumors [12–17]. PS exposure on tumor vessels is increased by irradiation induced apoptosis (Figure 2) [12, 23, 47]. We have confirmed externalization of PS on the luminal surface of vascular endothelium *in vivo* after radiation (Figure 3). At 6 hours after injection, externalization of PS was observed on the endothelium. Twenty-four hours after irradiation, mice were injected with a low dose of tissue factor and lipopolysaccharide (LPS) to induce thrombosis in GBM vasculature. The mechanism of thrombosis is that irradiation induces externalization of PS on the glioma vessel luminal surface. The externalised PS binds to tissue factor in blood circulation and triggers the coagulation cascade [48, 49]. They initially form a complex with factor VIIa. The factor VIIa-tissue factor complex catalyses the activation of factor IX and factor X, leading to the conversion of prothrombin to thrombin and thence the production of fibrin from fibrinogen. The fibrin thrombi cause vessel occlusion in the glioma vasculature (Figure 4). 

Irradiation of GBM vessels also possibly leads to thrombus formation in saline controls (Figure 4). Multiple interacting pathways contribute to this vascular shift toward the pro-thrombotic state, such as upregulation of von Willebrand factor (Figure 3) [50, 51] and increased externalization of phosphatidylserine on the cell membrane [15]. Under normal conditions, PS is confined to the internal leaflet of the plasma membrane [15]. PS asymmetry is maintained by a combination of activities: ATP-dependent translocase that moves phospholipids in both directions and a Ca^2+^-dependent scramblase that nonspecifically randomizes phospholipids across the bilayer [35]. Loss of asymmetry may be caused by inhibition of ATP-dependent translocase moving PS to the inner cell membrane or scramblase that displaces PS to the external membrane [15]. The exposure of PS on platelets allows the coordinated assembly of the tenase and prothrombinase complexes that lead to thrombin formation during clot deposition [49]; similar effects are likely to occur on cerebral endothelial cells with externalised PS. Radiation induces endothelial cell apoptosis. It is not clear from the current experiment to what extent apoptosis is responsible for the observed loss of PS asymmetry. Some irradiated vessels in the region of GBM in saline controls were observed externalization of PS on all of the endothelial cells. It is unlikely that all of the endothelial cells in the current experiment are undergoing apoptosis. While apoptosis may be responsible for some of PS externalization, other mechanisms, such as translocase stimulation, are likely to be involved [52].

Endothelial cell loss by irradiation induced apoptosis exposes the raw thrombogenic components of the subendothelium, such as collagen, basement membrane, tissue factor, von Willebrand factor, microfibrils, and fibronectin [53], leading to platelet adhesion. Within the irradiated GBM samples, capillaries were often seen that had no positive staining for von Willebrand factor in the intima, indicating loss of the endothelium. The underlying subendothelium, rich in tissue factor, was exposed to the vessel lumen. Some of these vessels were occluded by thrombi rich in von Willebrand factor. Exposure of subendothelial tissue factor appears to be important in the induction of thrombosis in GBM vessels. The primary function of tissue factor is the initiation of the extrinsic coagulation pathway.

Compared with our previous study in a rat model of arteriovenous malformations [18], significant higher rate of thrombosis was observed in the capillaries and medium sized vessels of GBM mice. The possible explanations are the following.

- Lower dose of irradiation was applied in the current protocol, which resulted in a higher viability of vascular endothelial cells. Therefore, a greater proportion of endothelial cells was involved in the thrombotic process.
- There are differences in the intrinsic characteristic between GBM mouse model and a rat model of arteriovenous malformations. The latter was surgically created by an anastomosis of the left common carotid artery to the left external jugular vein [18].
- There are structural and biological differences between cerebral vasculature in GBM mouse model and the external jugular vein in the rat model of arteriovenous malformations. For example, the former is rich in capillaries, and the latter mainly consists of branches that were dilated due to increased blood flow since a carotid-to-jugular anastomosis. The former has the brain-blood barrier and the latter without the brain-blood barrier.

Our data obtained from *in vivo* experiments provide strong evidence that vascular targeting approach can improve life expectancy and life quality of GBM mice (Figure 5). A combination of radiosurgery and soluble tissue factor plus lipopolysaccharide can not only prevent animal weight loss caused by GBM but also significantly increases animal body weight over a period of 30 days. In addition, this treatment improves animal quality of life, prolongs animal survival time, and reduces mortality to 20%. Such a body of evidence suggests that increased invasiveness of the tumor is unlikely in the posttreatment group using a combination of radiosurgery and soluble tissue factor plus lipopolysaccharide compared to the controls using PBS and radiosurgery or lipopolysaccharide and radiosurgery. In contrast, 50% mortality rate in a combination of radiosurgery and soluble tissue factor group is most likely due to insufficient thrombosis in GBM vessels, suggesting that lipopolysaccharide plays a significant role in vascular targeting. As previously reported, lipopolysaccharide offers a multitude of prothrombotic and proinflammatory effects [18]. It directly stimulates the release of soluble tissue factor, von Willebrand factor, and vascular cell adhesion molecule-1 [18]. The dose of lipopolysaccharide used in this study was much lower than that used previously which caused complications such as endotoxic shock in rats [54–56]. Shock is induced by doses around 4 to 8 *μ*g/g [54–56], whilst in this study, only 0.1 *μ*g/g was used. At this low dose, the maximal thrombotic effects were achieved in murine tumor vasculature [57]. In this study, there was no evidence of lipopolysaccharide toxicity in the animals as demonstrated by minimal weight loss and no behavioral change after injection. Soluble tissue factor was given at similar dose-to-weight ratio as was previously used in the murine tumor [57]. Soluble tissue factor was administrated at the optimal timing after radiosurgery as was previously used in the murine tumor [57] and the rat model of arteriovenous malformations [18].

## 5. Conclusions 

This study demonstrates that radiosurgery enhanced treatment with soluble tissue factor and lipopolysaccharide can selectively induce thrombosis of the capillaries and medium sized vessels of glioblastoma, causing a selective shutdown of oxygen and nutrient supply to the tumor cells. This treatment improves the quality of life, increases life expectancy, and reduces mortality of glioblastoma mice. 

## Abbreviations

DMEM: Dulbecco's modified Eagle's medium
FIU: Fluorescence intensity units
GBM: Glioblastoma
H&E: Hematoxylin and eosin
LPS: Lipopolysaccharide
MTT: 3-(4,5-dimethylthiazol-2-yl)-2,5-diphenyltetrazolium bromide
PS: Phosphatidylserine
TF: Soluble tissue factor.
